# The Foreign Journals: Statistics

**Published:** 1841-07

**Authors:** 


### STATISTICS.

### *Account of the Petersburgh Foundling Hospital, and Establishment for bringing up Children.* By J. G. KOHL.

THIS establishment was founded by the Empress Catherine in 1770. Its extent was of course at first but limited, and even in 1790 there were only three hundred children in it. But since the commencement of the present century the number of its inmates has very greatly increased, and has varied at different times, from one thousand to five, ten, or even five and twenty thousand. In 1837 there were no less than 25,600 young people in this gigantic establishment, and the number of children admitted into it of late years has been from 1828 to 1829, between 3 and 4000; from 1830 to 1833, between 4 and 5000; and from 1835 to 1837, between 5 and 7000.

The admission of children is perfectly unrestricted; every one that is brought is taken in, and without further ado received into the establishment, and the government, so far from setting a limit to the admissions, has rather taken care to provide for the wants of the charity with extraordinary liberality. The original foundation by Catherine was, in proportion to the present means of the Institution, extremely small; but it has been increased by munificent presents from private individuals, and still larger gifts from Alexander, Paul, and Nicholas; and it is now one of the richest charities in Russia, and has many millions (of rubles) invested in houses. To these sources of income Alexander added the revenue derived from the manufacture of cards, and that from the *Lombard* (or general establishment for pawning, *Mont de Piété*), which, in consequence of the great fluctuations of private property in Petersburg, is an establishment of enormous extent. Thus it is that every year six or seven hundred millions of rubles are available for the Foundling-Hospital, and pass through the hands of its director. The maintenance of the whole establishment now costs 5,200,000 rubles a year; in 1837, two millions was spent in building, and 300,000 in the erection of a church for the inmates.

The principal establishments are at Petersburg and at Gatschina, but their benefits are extended over the whole neighbourhood of Petersburg. In the latter city are the chief buildings for the reception of the children of both sexes, and for the care of them during the first six weeks; after which time, when they can bear removal, they are sent to people in the villages and towns within a circuit of 130 wersts from the city, with whom they remain till they are six years old. At this age the girls are brought back to Petersburg for further education, and the boys are sent for the same purpose to the establishment at Gatschina. When their education is completed, they are all permitted to leave the Institution, and are left perfectly free to follow their own choice of an occupation, or are sent to the business for which, according to their capacities, they have been prepared.

Six or seven hundred nurses are kept at the Institution, and twelve physicians, mostly Germans, officiate in the hospitals attached to it, and have the charge of examining and frequently visiting the children that are sent into the provinces. A lying-in hospital is also attached to the Foundling, and is based on the most liberal principles, every one that wishes being admitted without restriction. Complete secrecy however is maintained over this part of the establishment, and it is open to none but those who are actually attached to it.

At the Foundling the door of the little receiving room is open day and night throughout the year, and an inspectress, with several female assistants, is constantly in attendance. A thick book is kept in which the children, amounting to fifteen or twenty daily, are entered. The only question asked on receiving each of them is, whether it has been baptized, and whether it has a name. If this be the case, the child is entered in its own name; but if not, it is entered with a certain number attached to it. The women come in the greatest numbers at the close of the evening, with their children wrapped in cloths, and their attendance is more numerous in fine than in bad weather, and in summer than in winter, but most of all in the spring. We were at the Institution at one o'clock in the day, and at that time seven new inmates had been received, whose numbers we saw entered in fresh ink, 2310 to 2317. It not unfrequently happens that when the mother undoes the clothes, she finds her child dead; they are then not received, but are sent to be examined by the police. The living children however are at once received without reference to their origin; they are all baptized and admitted into the true church, and after six weeks are sent into the provinces. A fourth of them however die within these six weeks, and half of those that are sent away die within the next six years, so that only about a third of those that are received survive their sixth year, while that of the children brought up at their own homes fully half survive that time. A great proportion of this heavy mortality depends on the great distances through which they are carried to and from the establishment, for many of them come from remote parts of Russia, (indeed from all parts except those from which the superfluous children are sent to Moscow), and many are half dead before they arrive. In 1836, for example, there arrived on one day a child from Kischeneff, in Bessarabia, and another from Tobolsk, in Siberia, both of which places are about 250 miles from Petersburg. How many therefore must die even before they arrive at their destination! *Casper's Wochenschrift. Marz* 13, 1841.

---

